# Study of membrane potential in T lymphocytes subpopulations using flow cytometry

**DOI:** 10.1186/1471-2172-9-63

**Published:** 2008-11-03

**Authors:** Fernanda Mello de Queiroz, Cristiano G Ponte, Adriana Bonomo, Rosane Vianna-Jorge, Guilherme Suarez-Kurtz

**Affiliations:** 1 Molekulare Biologie Neuronaler Signale, Max-Planck-Institut für Experimentelle Medizin, Hermann-Rein-Strasse 3, 37075 Göttingen, Germany; 2 Divisão de Farmacologia, Coordenação de Pesquisa, Instituto Nacional de Câncer, Rio de Janeiro, Brazil; 3 Centro Federal de Educação Tecnológica de Química do Rio de Janeiro, Rio de Janeiro, Brazil; 4 Divisão de Medicina Experimental, Coordenação de Pesquisa, Instituto Nacional de Câncer, Rio de Janeiro, Brazil; 5 Instituto de Microbiologia Professor Paulo de Góes, Universidade Federal do Rio de Janeiro, Rio de Janeiro, Brazil; 6 Departamento de Farmacologia Básica e Clínica, Universidade Federal do Rio de Janeiro, Rio de Janeiro, Brazil; 7 Departamento de Bioquímica Médica, Universidade Federal do Rio de Janeiro, Rio de Janeiro, Brazil

## Abstract

**Background:**

Ion channels are involved in the control of membrane potential (ψ) in a variety of cells. The maintenance of ψ in human T lymphocytes is essential for T-cell activation and was suggested to depend mostly on the voltage-gated Kv1.3 channel. Blockage of Kv1.3 inhibits cytokine production and lymphocyte proliferation *in vitro *and suppresses immune response *in vivo*. T lymphocytes are a heterogeneous cell population and the expression of Kv1.3 varies among cell subsets. Oxonol diBA-C4-(3) was used to determine ψ by flow cytometry. The presence of distinct T cell subsets was evaluated by immunophenotyping techniques and the contribution of Kv1.3 channels for the maintenance of ψ was investigated using selective blockers.

**Results:**

The distribution of ψ in T lymphocytes varied among blood donors and did not always follow a unimodal pattern. T lymphocytes were divided into CD3^+^/CD45RO^- ^and CD3^+^/CD45RO^+ ^subsets, whose peak channel values of ψ were -58 ± 3.6 mV and -37 ± 4.1 mV, respectively. MgTX (specific inhibitor of Kv1.3 channels) had no significant effect in the ψ of CD3^+^/CD45RO^- ^subsets but depolarized CD3^+^/CD45RO^+ ^cells to -27 ± 5.1 mV.

**Conclusion:**

Combination of optical methods for determination of ψ by flow cytometry with immuophenotyping techniques opens new possibilities for the study of ion channels in the biology of heterogeneous cell populations such as T lymphocyte subsets.

## Background

Electrical potential differences are generated across the cytoplasmic membranes of animal cells by concentration gradients of ions such as Na^+^, K^+^, Cl^- ^and H^+^. The maintenance of membrane potential (ψ) depends on ion channels, ion pumps and eletrogenic transporters. Ion channels also regulate various cell functions such as: electrical excitability of myocytes and neurons [1], cell proliferation [2-4] and hormone secretion [5,6]. The study of ψ variations require the use of electrophysiological methods [1,7], the patch-clamp being the gold-standard technique [7], because it allows detailed biophysical characterization of ion channels [8,9] and, combined with pharmacological tools, the study of their contribution to ψ [9,10]. However, patch-clamp analysis is restricted to one cell at a time, limiting its application for the study of large and heterogeneous cell populations. Optical methods for the determination of ψ were introduced by Cohen et al. [11] and are an alternative for the study of ψ variations in a large number of cells within a reasonably short period of time. These optical methods are based on the use of fluorescent dyes, which respond to membrane polarity stimuli causing changes in fluorescence [12]. Combination of optical methods for the measurement of ψ with flow cytometry (Fluorescence Activated Cell Sorter – FACS) techniques opens new possibilities for the study of ion channels in the biology of heterogeneous cell populations.

Human T lymphocytes are a good example of a heterogeneous cell population in which the study of ion channels and their contribution for ψ is of great interest. The activation of T lymphocytes during the immune response requires continuous Ca^2+ ^influx across the plasma membrane [13,14]. The voltage-gated K^+ ^channel, Kv1.3 [8,15] and the Ca^2+^-activated-K^+ ^channel, KCa3.1 modulate calcium influx by regulating the ψ and providing electrical driving force for continuous Ca^2+ ^entry [8,16]. While KCa3.1 blockers are able to prevent proliferation in mitogen-activated lymphocytes [16], blockage of Kv1.3 channels by specific inhibitors, such as margatoxin (MgTX) prevent proliferation in resting T cells. Blockage of Kv1.3 channels causes a depolarization of the ψ leading to a reduction in the intracellular Ca^2+ ^concentration [8,16]. As a consequence, cytokine production and cell proliferation are inhibited [15], which attenuates immune response *in vivo *[2]. Data in the literature regarding expression of Kv1.3 and control of ψ were obtained with path-clamp techniques on isolated T cells activated *in vitro *[17-19]. Peripheral T cells, however, are composed of non-activated (naive) T cells, pre-activated T blasts and memory T cells. Data obtained by optical methods estimate that the ψ of peripheral T cells vary between -70 and -45 mV [20-22], suggesting that different subsets of T cells present in peripheral blood have distinct ψ.

The membrane potential-sensitive fluorescent dye oxonol (diBA-C4-(3) was chosen due to advantages over other dyes: i) it is non-cytotoxic, ii) not shown to block ion channels and iii) it is not extruded by the glycoprotein efflux pump [23,24]. In the present work we combine oxonol with FACS-immunophenotyping techniques in order to characterize the ψ in specific sub-populations of human T lymphocytes [25]. We use specific inhibitors of potassium channels to evaluate the role of voltage-gated K^+ ^channels in controlling the ψ in naive and in memory T cells.

## Results

### Validation of FACS estimates of ψ

The calculation of ψ was based on the Nernst equation: ψ = RT/F*ln(Ox_i_/Ox_e_), where R is the universal gas constant, T is the absolute temperature, F is the Faraday's constant and Ox_i _and Ox_e _are the internal and external concentrations of oxonol, respectively. The calibration curve was determined using different concentrations of extracellular oxonol. Since the external and internal concentrations of the dye are equal when the ψ is the same (Ox_i _= Ox_e _*exp ψF/RT), one can assume a new calibration curve based on Ox_i_. The ratio Ox_i_/Ox_e _was calculated based on the acquisition of a fixed sample (ψ equal to 0 mV) using the same Ox_e _for both. Afterwards the ratio value of Ox_i_/Ox_e _was used to calculate the ψ based on the Nernst equation [26].

**Figure 1:**
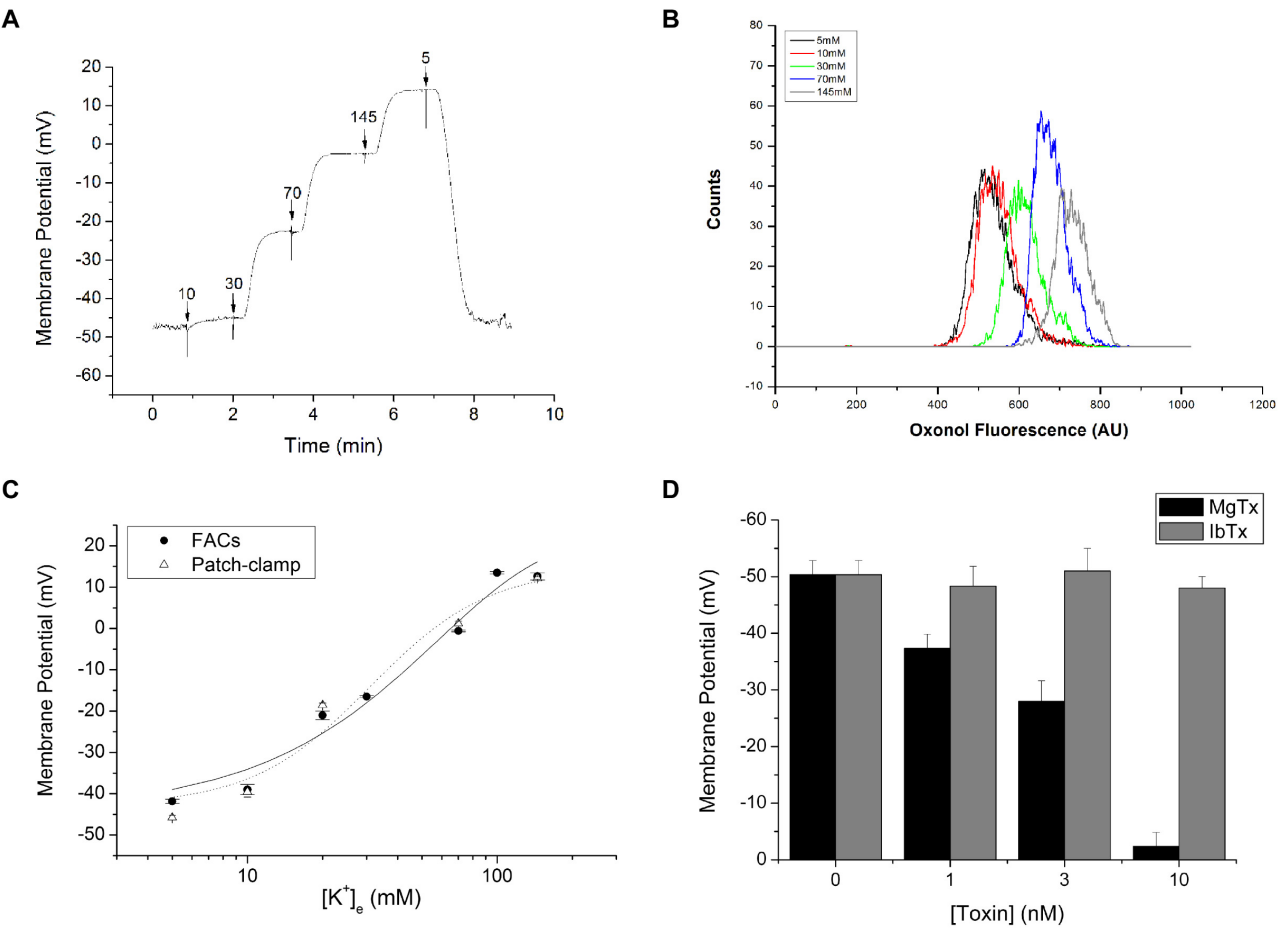
**Validation of ψ quantification by FACS**. Representative trace of a CHO-Kv1.3 cell treated with different concentrations of [K^+^]_e _recorded by patch-clamp (A) or by FACS (B). (C) Dependence of absolute ψ of CHO-Kv1.3 cells on the [K^+^]_e _determined by FACS (●) and patch-clamp (Δ) techniques. Data of ψ against increasing [K^+^]_e _(5–145 mM) were plotted and fitted with a Boltzman equation. Data represent mean ± SE of four experiments. (D) CHO-Kv1.3 cells were treated with different concentrations (1, 3 and 10 nM) of MgTx (black bars) or IbTx (grey bars) and their ψ was measured by FACS. Data represent mean ± SD of three experiments.

We characterized the variation of ψ in Kv1.3-transfected CHO (CHO-Kv1.3) cells exposed to different concentrations of extracellular K^+ ^([K^+^]_e _= 5–145 mM). Figure 1A and 1B shows the values of ψ measured by patch-clamp and the oxonol fluorescence by FACS, respectively. The values of ψ measured by either FACS or by patch-clamp were compared (fig 1C). The curves show an overlay in the range of -40 to +10 mV, indicating the reliability of ψ measurements obtained with FACS.

Our aim was also to test if this technique could discriminate between distinct ψ generate by different concentration of pharmacological blockers. CHO-Kv1.3 cells were treated with two toxins: MgTx (black bars) or iberiotoxin (IbTx; grey bars) and the ψ was measured by FACS (fig. 1D). The IbTx was chosen as a control blocker for the Kv1.3 channel, since it is the most potent and high-affinity blocker for the high-conductance calcium-activated potassium channel (BK_Ca_) and it has none or low affinity for the Kv1 channels [27]. Addition of MgTx depolarized CHO-Kv1.3 cells on a dose-dependent manner shifting ψ from -50.3 ± 2.5 to -3.6 ± 2.5 when the concentration of 10 nM was used. IbTx, which is a selective blocker of the BK_Ca _channel [28], had no effect on the ψ of CHO-Kv1.3 cells.

**Figure 2:**
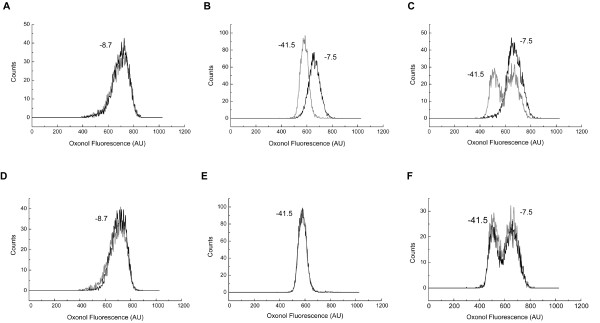
**FACS measurement of ψ distinguishes CHO-Kv1.3 from CHO cells**. Histograms of oxonol fluorescence in CHO (A, D), CHO-Kv1.3 (B, E) and a combination of CHO and CHO-Kv1.3 (C, F) untreated (gray lines) or treated (black lines) with MgTX (A, B, C) or IbTX (D, E, F). The ψ values in mV are depicted. Data are representative of four experiments.

In order to evaluate the ability of this method to distinguish cell populations with different ψ, we used two established cell lines, CHO and CHO-Kv1.3 [29]. Figure 2 shows a representative experiment, illustrating the distribution of ψ in CHO (panels A and D), CHO-Kv1.3 (panels B and E), and in a mixed population containing CHO and CHO-Kv1.3 cells (panels C and F). The peak channel of ψ in CHO-Kv1.3 cells is shifted leftwards as compared to CHO cells, and the two cell subsets can be visually distinguished when mixed in the same sample (fig. 2A–C gray lines). The mean values of ψ were -8.7 ± 2.3 mV for CHO and -41.5 ± 1.3 mV for CHO-Kv1.3 (N = 4 different experiments) which are in accordance with data published by Defarias et al. [29]. Addition of 10 nM MgTX (fig. 2A–C, black lines) had no effect on the distribution of ψ in CHO cells (fig. 2A), but it depolarized CHO-Kv1.3 cells (fig. 2B) by shifting the peak channel value of ψ to -7.5 ± 1.9 mV (P 0.0001). In the presence of MgTX, CHO and CHO-Kv1.3 subsets were indistinguishable (fig. 2C). Addition of 10 nM iberiotoxin (fig. 2D–F, black line) had no effect on the distribution of ψ of either CHO (fig. 2D) or CHO-Kv1.3 (fig. 2E) and, therefore, did not affect the ψ pattern of cell subsets when they are mixed (fig. 2F).

These results indicate that it is possible to characterize the ψ of different cell populations using FACS and to evaluate the contribution of ion channels for maintenance of ψ by using specific ion channel blockers.

### Distribution of ψ on peripheral blood lymphocytes

**Figure 3:**
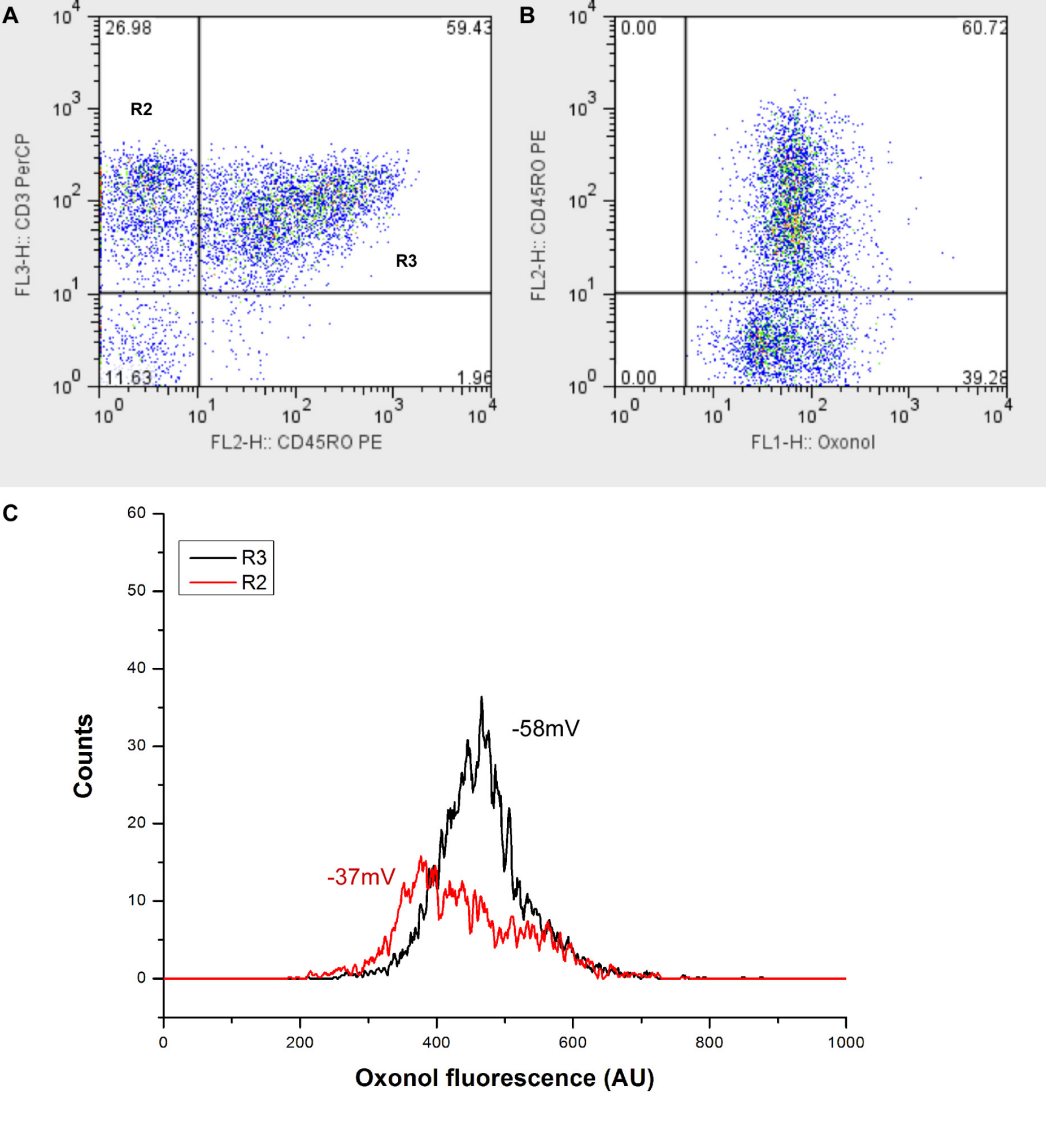
**Distinct ψ in different subsets of PBL**. (A) Dot-plot of control PBL stained with anti-CD3 (y-axis) and anti-CD45RO (x-axis). The CD3^+^/CD45RO^- ^and CD3^+^/CD45RO^+ ^sets are depicted in the upper left (R2) and upper right gate (R3), respectively. (B) Dot-plot of the CD45RO sub-population according to the oxonol fluorescence distribution. Numbers displayed inside the dot-plot represent the percentage of cells contained in each quadrant. (C) Histograms distributions of oxonol fluorescence in control PBL cells gated on R2 (red) or R3 (black). Numbers depicted represent the ψ values. Data are representative of six experiments.

Human mononuclear cells from peripheral blood (PBMC) were immunostained with CD3 and CD45RO mAb and loaded with oxonol in order to evaluate the ψ in peripheral blood lymphocytes (PBL). PBL were gated according to their physical characteristics and the patterns of CD3 and CD45RO were analyzed in Figure 3A. Two subsets of T lymphocytes (CD3^+ ^cells) can be identified in relation to the expression of CD45RO. Thus, CD3^+^/CD45RO^- ^are in the upper left gate (R2), whereas the CD3^+^/CD45RO^+ ^are in the upper right gate (R3). Figure 3B shows the CD45RO^+ ^subset according to the oxonol fluorescence distribution. The distribution of ψ in CD3^+ ^cells did not always follow a unimodal pattern, as the example illustrated in figure 3B. In two out of six blood donors, we obtained a bimodal distribution of ψ. Separation of CD3^+ ^cells according to the expression of CD45RO allowed characterization of ψ in the two cell subsets. Thus, CD3^+^/CD45RO^- ^cells were hyperpolarized in relation to CD3^+^/CD45RO^+ ^cells (fig. 3C), the peak channel values of ψ being -58 ± 3.6 mV and -37 ± 4.1 mV, respectively (P = 0.0087).

### Effects of MgTX or high [K^+^]_e _on the ψ of PBL

**Figure 4:**
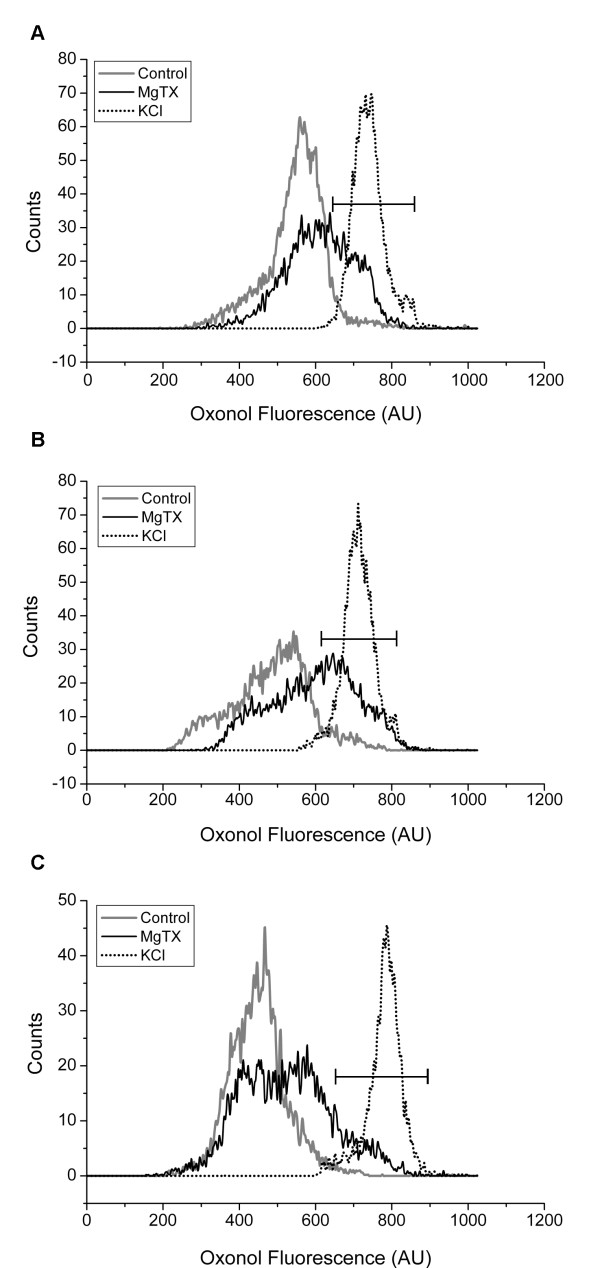
**Effects of MgTX or high [K^+^]_e _on CD3^+ ^cells**. (A, B, C) Histogram distributions of oxonol fluorescence in human T lymphocytes (CD3^+^) from three different donors in control (gray lines) or treated with MgTX (full line) or high [K^+^]_e _(dashed line). The black marker indicates depolarized cells as determined by [K^+^]_e _treatment. Data are representative of six experiments.

Figure 4 shows the histograms of ψ distribution obtained with CD3^+ ^cells from three different donors (A, B and C) in control (gray line) and upon treatment with either 10 nM MgTX (black line) or high [K^+^]_e _(145 mM; dotted line). The three control CD3^+ ^samples represent the distinct distribution patterns of ψ. Thus, donors A and C showed a unimodal pattern on the control trace, whereas donor B showed a bimodal pattern. Addition of high [K^+^]_e _depolarized all the samples to approximately 0 mV (peak channel evaluation) and generated a unimodal pattern in all histograms studied. In contrast, addition of MgTX produced diverse degrees of response, causing partial depolarization, which broadened the ψ distribution (Fig. 4A) or generated a bimodal pattern (Fig. 4C). Percentage of cells which depolarized upon treatment with MgTx and overlapped with the histogram acquire after treatment with high [K^+^]_e _were determined. Addition of MgTx did not depolarize all population when compared with the high [K^+^]_e _treatment; it rather depolarized a small part (black marker) which accounts for 27, 39 and 12% of CD3^+ ^cells from the different blood donors (fig. 4A,B and 4C, respectively).

In order to characterize the distinct degrees of depolarization upon exposure to MgTX in activated T lymphocytes, we investigated the presence of CD45RO^- ^and CD45RO^+ ^cells in the different samples and evaluated the sensitivity of each cell subset to MgTX. The proportion of CD45RO^- ^and CD45RO^+ ^varied among donors and the individuals with higher amounts of CD45RO^+ ^cells among T lymphocytes (N = 4) showed significant depolarization upon treatment with MgTX (from -45 to -15 mV, P = 0.0025), whereas those with low proportion of CD45RO^+ ^cells (N = 2) were not affected by MgTX exposure (data not shown).

**Figure 5:**
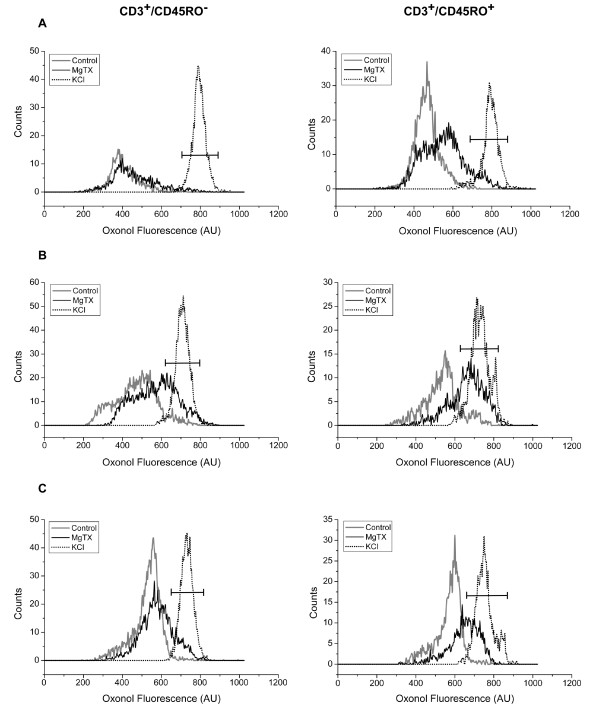
**Effects of MgTX or high [K^+^]_e _on subsets of CD3^+ ^cells**. Histograms distribution of oxonol fluorescence in CD3^+^/CD45RO^- ^and CD3^+^/CD45RO^+ ^untreated cells (gray lines) and treated with MgTX (full lines) or high [K^+^]_e _(dashed lines). The proportions of CD3^+^/CD45RO^- ^and CD3^+^/CD45RO^+ ^were 30% and 60% (A), 60% and 30% (B) and 45% and 35% (C) for the CD45RO^- ^and CD45RO^+ ^populations of the total CD3^+^, respectively. The black marker indicates depolarized cells as determined by [K^+^]_e _treatment. Data are representative of six experiments.

In view of these results, we analyzed the distribution of ψ and the effects of MgTX in CD45RO^- ^and CD45RO^+ ^subsets. Figure 5 shows the results obtained in three out of six donors (panels A, B and C), which illustrate the different patterns and degrees of response. The CD45RO^- ^and CD45RO^+ ^subsets from each donor are shown in left and right panels, respectively. The CD45RO^+ ^subsets presented a unimodal distribution of ψ in all donors studied. In contrast, CD45RO^- ^subsets had a more variable distribution of ψ, with a bimodal pattern being seen in two out of six donors (panel B-left shows an example). Addition of high [K^+^]_e _(dotted line) depolarized all cell subsets. In contrast, addition of MgTX had no significant effect in CD45RO^- ^subsets (P = 0.15, N = 6) but depolarized CD45RO^+ ^cells, shifting the peak channel value of ψ from -37.2 ± 4.1 mV to -26.7 ± 5.1 mV (P = 0.0025, N = 6). By comparing CD45RO^- ^and CD45RO^+ ^subsets that depolarized at the same extent as high [K^+^]_e _treatment (marker), we had always 2-fold increase of percentage of cells in the later subset for each donor. Percentage of cells was 5 and 13%, 29 and 64% and 17 and 37% (CD45RO^- ^and CD45RO^+^, respectively; fig. 5A,B and 5C).

## Discussion

The main goal of the present study was to combine the methodology described by Krasznai et al. [26] with other FACS techniques and the use of specific ion channel blockers in order to study the ψ of T lymphocytes. CHO and CHO-Kv1.3 cells are well-established cell lines, widely used in electrophysiology [29-32]. The mean values of ψ determined by FACS in these cell lines are in agreement with the data from electrophysiological studies [29]. The dispersion of ψ values was higher for CHO cells (-70 to +30 mV) than for CHO-Kv1.3 cells (-70 to -30 mV). CHO cells have their ψ controlled partly by chloride channels and partly by cation channels [29]. The transfection of Kv1.3 to CHO cells sets the resting ψ to values close to -50 mV, similarly to what is observed in human peripheral T lymphocytes [8,33]. The narrower dispersion of ψ values in CHO-Kv1.3 cells as compared to CHO cells corroborates the idea that Kv1.3 is the main responsible for the control of ψ in these cells. This is confirmed by the fact that MgTX, but not IbTX, depolarizes CHO-Kv1.3 cells and enlarges the dispersion of ψ to values similar to those of CHO cells (-60 to +30 mV, fig. 1C).

PBMC are a heterogeneous population, composed of T and B lymphocytes, NK cells and monocytes. Expression of Kv1.3 has been reported in T and B lymphocytes and in monocytes/macrophages [34]. In the present study, we evaluate the ability of Kv1.3 channel to control the ψ in different T lymphocyte subsets, allying an optical method for determination of ψ by FACS with immunophenotyping techniques. Human T lymphocytes were identified by the expression of CD3 and subdivided into CD3^+^/CD45RO^- ^and CD3^+^/CD45RO^+ ^cells. Thus, CD3^+^/CD45RO^- ^cells include naive and recently activated T lymphocytes, whereas CD3^+^/CD45RO^+ ^correspond to memory T lymphocytes [25]. The proportion of CD3^+^/CD45RO^- ^and CD3^+^/CD45RO^+ ^varied among different donors (see figure 4) reflecting the dynamic regulation of the immune system.

When analyzed together, CD3^+ ^cells showed different patterns of ψ distribution and variable sensitivity to MgTX, suggesting that the T lymphocyte subsets have different ψ and are differently regulated by Kv1.3. Accordingly, it has been recently shown that naive and memory T cells have differences in the expression of Kv1.3 and KCa3.1 channels. Thus, naive cells express about 200–400 Kv1.3 channels along with 8–10 KCa3.1 channels per cell, whereas memory T cells may have up to 1800 Kv1.3 channels/cell [35]. The markers for discriminating naive and memory T cells used in this study are different from the ones published by Wulff et al. [35], nevertheless there is an overlap between the subsets studied. In view of this channel distribution, it would be expected that CD3^+^/CD45RO^+ ^cells, were hyperpolarized in relation to CD3^+^/CD45RO^- ^cells, unlike the results shown in figure 3. However, CD3^+^/CD45RO^- ^cells include naive and recently activated T blasts [25], and the latter express 500–600 KCa3.1 channels, which have been shown to shift the ψ to -80 mV [36]. Thus, the broad distribution of ψ (sometimes with a bimodal pattern) within CD3^+^/CD45RO^- ^cells may be due to the presence of activated T blasts in the peripheral blood of some donors. Accordingly, CD3^+^/CD45RO^- ^cells were not significantly depolarized by MgTX, suggesting a minor role of Kv1.3 channels among these cells.

Memory T cells comprehend two sub-populations, which have been classified as central memory (TCM) and effector memory (TEM) cells, based on their homing potentials and effector functions [37]. These two memory cell subsets differ in relation to the expression of Kv1.3 and KCa3.1 channels. TCM cells have 250–300 Kv1.3 channels/cell and up-regulate KCa3.1 from 20 to 500–600 channels/cell following activation, whereas TEM cells up-regulate Kv1.3 channels to 1500–1800 channels/cell and down-regulate KCa3.1 to 50–100 channels/cell after repeated activation [35]. In the present study, we did not distinguish these two memory T cell subsets and we are aware that further experiments are necessary to study these T cells subsets and examine the effect of Kv1.3 and KCa3.1 channels blockers. Nonetheless, our results corroborate the notion that Kv1.3 channels are the main responsible for the control of ψ among memory T cells, since MgTX caused significant depolarization. It is noteworthy, though, that the depolarization was partial in some cases (see figure 5A), suggesting the presence of a less sensitive subset (possibly composed of TCM cells). The correlation between a specific channel and its ability to maintain the resting potential of a particular population of cells requires the use of more specific blocker since many of the available pharmacological channel blockers target more than one channel. Nevertheless, we can suggest that the Kv1.3 channel is involved in the regulation of ψ from the CD3^+^/CD45RO^+ ^subset of T lymphocytes (fig. 5).

The fact that Kv1.3 channel is functionally restricted regarding tissue distribution together with the improvement of experimental autoimmune encephalomyelitis [38] and delayed type hypersensitivity in animal models without causing obvious side effects has made Kv1.3 an interesting therapeutic target [2,39,40]. A rapid screening of new ion channel blockers and the determination of the exact subset of cells affected by these blockers would be of great interest in the development of new immunossupressive therapies.

## Conclusion

In summary, our results indicate that FACS determination of ψ can be used for identification of ψ heterogeneity among cell populations. Combination of this method with other FACS techniques could also be used for determination of ψ in different cell cycle phases, developmental stages or activation patterns and for rapid screening of new ion channel blockers. This represents a new strategy for studying the role of ion channels in cell growth and differentiation of normal and tumoral cells.

## Methods

### Cells

CHO cells were obtained from Rio de Janeiro Cell Bank (PABCAM, Federal University, Rio de Janeiro, RJ, Brazil) and CHO-Kv1.3 cells [29] were a kind gift from Dr. Maria L. Garcia (Merck & Co., Rahway, NJ, USA). Both cell lines were maintained in α-Minimum Essential Medium (Gibco-BRL – Life Technologies, Inc., Grand Island, NY, USA) supplemented with 10% (v/v) heat-inactivated bovine fetal serum (Gibco), 60 mg/L penicillin (Sigma Chemical Co., St Louis, MO, USA), 100 mg/L streptomycin (Sigma) 2.4 g/L HEPES (Sigma). Cultures were grown in a humidified incubator at 37°C, 5% CO_2_.

PBMC were isolated from the peripheral blood of healthy donors by centrifugation on a Ficoll gradient. PBMC were washed and incubated with RPMI 1640 medium (Sigma) supplemented with 10% (v/v) heat-inactivated bovine fetal serum (Gibco), 60 mg/L penicillin (Sigma), 100 mg/L streptomycin (Sigma) for 30 minutes (37°C, 5% CO_2 _humidified atmosphere). The study was evaluated by the National Cancer Institute (INCa-Brazil/RJ) Ethical Committee and the informed consent of all participating subjects was obtained.

Samples were fixed with ice-cold 2% formaldehyde and kept at 40°C for 60 minutes. Cells were washed with PBS and kept at room temperature before the measurements.

### Reagents

Bis(1,3-dibutylbarbituric acid(5)) trimethine oxonol (diBA-C4-(3)), obtained from Molecular Probes (Invitrogen, Carlsbad, CA, USA), was dissolved in DMSO and stored in aliquots (1 mM) at -20°C, under protection from light. Aliquots were added to the cell suspensions to yield the desired final concentration (5–1500 nM). The oxonol concentration of 100 nM was used for the membrane potential measurements. The DMSO final concentration (0.1% (v/v)) had no effect on cell viability. Stock solutions of MgTX (kindly provided by Dr. Maria L. Garcia, Merck & Co.) and IbTX (Alamone Labs, Jerusalem, Israel) were prepared in a saline solution with 100 mM NaCl, 20 mM Tris-HCl (pH 7.4), and 0.1% (w/v) BSA. All other reagents were of analytical grade.

### Electrophysiology

Membrane potentials of CHO-Kv1.3 cells were measured by whole-cell patch-clamp recordings obtained with an EPC-7 amplifier (Axon Instruments, Foster City, CA, USA) in current clamp mode with a 2 KHz analogical filter. The signals were digitized at 5 KHz (interface DigiData 1200 – Axon Instruments) and analyzed with the software pClamp 6.0 (Axon Instruments). Membrane voltages were corrected for liquid junction potentials before patching the cell at the voltage clamp mode. Pipettes with resistances of 3–6 M, were pulled from thin-walled borosilicate glass capillaries (Rochester Scientific Co. Inc., Rochester, NY, USA), and filled with a solution containing (in mM): 140 KCl, 2 MgCl2, 0.123 CaCl2, 0.2 K2-EGTA, 10 Hepes (pH 7.4). The extracellular medium, designated PSS (physiological saline solution), contained (in mM): 140 NaCl, 5 KCl, 2 MgCl2, 1 CaCl2, 10 HEPES (pH 7.4). High K^+ ^solutions (10 – 145 mM) were obtained by isotonic replacement of NaCl with KCl in the PSS. Experiments were performed at room temperature (22–25°C). The liquid junction potential correction was performed using the software JPCalc [41].

### Flow Cytometry (FACS)

We used the method described by Krasznai et al. [26] for determination of ψ. The phosphate buffer solution (PBS) was replaced by the PSS, which is the standard solution in all experiments of electrophysiology in our laboratory. The method was validated using the PSS in CHO cells and in human lymphocytes.

PBMC were labeled with a PercP-conjugated anti-human CD3 or PE-conjugated CD45RO mouse antibody (Pharmigen, San Diego, CA, USA), Fc receptor being blocked with normal mouse serum (1:50) in PBS buffer. After a 20 minute-incubation with the antibody at 4°C, cells were washed with PSS kept at room temperature and re-suspended at a concentration of 10^6 ^ml-1.

Measurements were carried out at room temperature, using a Becton Dickison FACScan flow cytometer and data were analyzed using Cell Quest or FlowJo program. Forward-scatter (FSC) and side-scatter (SSC) lights were used for gating of data acquisition. Non-viable cells were identified with propidium iodide (Sigma), and were excluded from analysis. All samples were excited with the 488 nm line and oxonol, PE and PercP fluorescence emission were captured at 530/30 nm, 585/42 nm and 670 nm long pass, respectively. The calculated values of ψ within a cell subset are presented in histogram distributions and the peak channels were used for comparative analysis. All the experiments were performed at least four times and the data are presented as mean ± standard error.

### Statistical Analysis

Unpaired t test was performed for comparison of the values of ψ between cell subsets and paired t test was used for comparisons of ψ after different treatments within a given cell subset. The software GraphPad Prism version 4 was applied for the analysis.

## Authors' contributions

FMQ carried out all flow cytometry experiments, and drafted the manuscript. CGP carried out all electrophysiology experiments. AB contributed with intellectual expertise to the flow cytometry data and drafted the manuscript. RVJ and GK helped to draft the manuscript. All authors read and approved the final manuscript.

**Table 1:** The ψ of T lymphocytes subsets

	**ψ (mV)**							
**Volunteers**	***1***	***2***	***3***	***4***	***5***	***6***	***Mean***	***SE***

**CD3**	55	66	40	60	45	50	52.7	3.9
**MgTx**	30	45	32	28	25	21	30.2	3.4
**high- [K^+^]_e_**	3	5	2	8	0	1	3.2	1.2
**CD45RO-**	60	66	50	70	47	55	58.0	3.7
**MgTx**	57	60	45	72	45	30	51.5	6.0
**high- [K^+^]_e_**	3	5	2	8	0	1	3.2	1.2
**CD45RO+**	39	45	30	52	25	32	37.2	4.1
**MgTx**	21	40	20	45	15	19	26.7	5.1
**high- [K^+^]_e_**	3	5	2	8	0	1	3.2	1.2

The ψ was obtained from the conversion of the peak channel value generate by the Cell Quest software for each volunteer.

## References

[B1] Hille B (2001). Ion Channels of Excitable Membranes.

[B2] Koo GC, Blake JT, Talento A, Nguyen M, Lin S, Sirotina A, Shah K, Mulvany K, Hora D, Cunningham P (1997). Blockade of the voltage-gated potassium channel Kv1.3 inhibits immune responses in vivo. J Immunol.

[B3] Nilius B, Wohlrab W (1992). Potassium channels and regulation of proliferation of human melanoma cells. J Physiol.

[B4] Wang L, Xu B, White RE, Lu L (1997). Growth factor-mediated K+ channel activity associated with human myeloblastic ML-1 cell proliferation. Am J Physiol.

[B5] Shibasaki T, Sunaga Y, Fujimoto K, Kashima Y, Seino S (2004). Interaction of ATP sensor, cAMP sensor, Ca2+ sensor, and voltage-dependent Ca2+ channel in insulin granule exocytosis. J Biol Chem.

[B6] Rossi NF (1995). Cation channel mechanisms in ET-3-induced vasopressin secretion by rat hypothalamo-neurohypophysial explants. Am J Physiol.

[B7] Hamill OP, Marty A, Neher E, Sakmann B, Sigworth FJ (1981). Improved patch-clamp techniques for high-resolution current recording from cells and cell-free membrane patches. Pflugers Arch.

[B8] Leonard RJ, Garcia ML, Slaughter RS, Reuben JP (1992). Selective blockers of voltage-gated K+ channels depolarize human T lymphocytes: mechanism of the antiproliferative effect of charybdotoxin. Proc Natl Acad Sci USA.

[B9] Zegarra-Moran O, Rasola A, Rugolo M, Porcelli AM, Rossi B, Galietta LJ (1999). HIV-1 nef expression inhibits the activity of a Ca2+-dependent K+ channel involved in the control of the resting potential in CEM lymphocytes. J Immunol.

[B10] Wang L, Zhou P, Craig RW, Lu L (1999). Protection from cell death by mcl-1 is mediated by membrane hyperpolarization induced by K(+) channel activation. J Membr Biol.

[B11] Cohen LB (1973). Changes in neuron structure during action potential propagation and synaptic transmission. Physiol Rev.

[B12] Waggoner AS (1979). Dye indicators of membrane potential. Annu Rev Biophys Bioeng.

[B13] Dolmetsch RE, Xu K, Lewis RS (1998). Calcium oscillations increase the efficiency and specificity of gene expression. Nature.

[B14] Lewis RS (2001). Calcium signaling mechanisms in T lymphocytes. Annu Rev Immunol.

[B15] Lin CS, Boltz RC, Blake JT, Nguyen M, Talento A, Fischer PA, Springer MS, Sigal NH, Slaughter RS, Garcia ML (1993). Voltage-gated potassium channels regulate calcium-dependent pathways involved in human T lymphocyte activation. J Exp Med.

[B16] Ghanshani S, Wulff H, Miller MJ, Rohm H, Neben A, Gutman GA, Cahalan MD, Chandy KG (2000). Up-regulation of the IKCa1 potassium channel during T-cell activation. Molecular mechanism and functional consequences. J Biol Chem.

[B17] Verheugen JA (1998). Elevation of intracellular Ca2+ in the physiologically relevant range does not inhibit voltage-gated K+ channels in human T lymphocytes. J Physiol.

[B18] Verheugen JA, Korn H (1997). A charybdotoxin-insensitive conductance in human T lymphocytes: T cell membrane potential is set by distinct K+ channels. J Physiol.

[B19] Rader RK, Kahn LE, Anderson GD, Martin CL, Chinn KS, Gregory SA (1996). T cell activation is regulated by voltage-dependent and calcium-activated potassium channels. J Immunol.

[B20] Wilson HA, Chused TM (1985). Lymphocyte membrane potential and Ca2+-sensitive potassium channels described by oxonol dye fluorescence measurements. J Cell Physiol.

[B21] Grinstein S, Smith JD (1990). Calcium-independent cell volume regulation in human lymphocytes. Inhibition by charybdotoxin. J Gen Physiol.

[B22] Aszalos A (1991). Cyclosporin elicits a non-responsive state and a shift in K+ fluxes in the early phase of activation of human lymphocytes with anti-CD3. Eur J Pharmacol.

[B23] Epps DE, Wolfe ML, Groppi V (1994). Characterization of the steady-state and dynamic fluorescence properties of the potential-sensitive dye bis-(1,3-dibutylbarbituric acid)trimethine oxonol (Dibac4(3)) in model systems and cells. Chem Phys Lipids.

[B24] Shapiro HM (2000). Membrane potential estimation by flow cytometry. Methods.

[B25] Dutton RW, Bradley LM, Swain SL (1998). T cell memory. Annu Rev Immunol.

[B26] Krasznai Z, Márián T, Balkay L, Emri M, Trón L (1995). Flow cytometric determination of absolute membrane potential of cells. J Photochem Photobiol B.

[B27] Garcia M, Galvez A, Garcia-Calvo M, King VF, Vazquez J, Kraczorowski GJ (1991). Use of toxins to study potassium channels. J Bioenerg Biomembr.

[B28] Galvez A, Gimenez-Gallego G, Reuben JP, Roy-Contancin L, Feigenbaum P, Kaczorowski GJ, Garcia ML (1990). Purification and characterization of a unique, potent, peptidyl probe for the high conductance calcium-activated potassium channel from venom of the scorpion Buthus tamulus. J Biol Chem.

[B29] Defarias FP, Stevens SP, Leonard RJ (1995). Stable expression of human Kv1.3 potassium channels resets the resting membrane potential of cultured mammalian cells. Receptors Channels.

[B30] Choi JS, Hahn SJ, Rhie DJ, Yoon SH, Jo YH, Kim MS (1999). Mechanism of fluoxetine block of cloned voltage-activated potassium channel Kv1.3. J Pharmacol Exp Ther.

[B31] Felix JP, Bugianesi RM, Schmalhofer WA, Borris R, Goetz MA, Hensens OD, Bao JM, Kayser F, Parsons WH, Rupprecht K (1999). Identification and biochemical characterization of a novel nortriterpene inhibitor of the human lymphocyte voltage-gated potassium channel, Kv1.3. Biochemistry.

[B32] Hahn SJ, Wang LY, Kaczmarek LK (1996). Inhibition by nystatin of Kv1.3 channels expressed in Chinese hamster ovary cells. Neuropharmacology.

[B33] Deutsch CJ, Holian A, Holian SK, Daniele RP, Wilson DF (1979). Transmembrane electrical and pH gradients across human erythrocytes and human peripheral lymphocytes. J Cell Physiol.

[B34] Aiyar J (1999). Potassium channels in leukocytes and toxins that block them: structure, function and therapeutic implications. Persp Drug Disc Desig.

[B35] Wulff H, Calabresi PA, Allie R, Yun S, Pennington M, Beeton C, Chandy KG (2003). The voltage-gated Kv1.3 K(+) channel in effector memory T cells as new target for MS. J Clin Invest.

[B36] Cahalan MD, Wulff H, Chandy KG (2001). Molecular properties and physiological roles of ion channels in the immune system. J Clin Immunol.

[B37] Sallusto F, Lenig D, Förster R, Lipp M, Lanzavecchia A (1999). Two subsets of memory T lymphocytes with distinct homing potentials and effector functions. Nature.

[B38] Beeton C, Barbaria J, Giraud P, Devaux J, Benoliel AM, Gola M, Sabatier JM, Bernard D, Crest M, Béraud E (2001). Selective blocking of voltage-gated K+ channels improves experimental autoimmune encephalomyelitis and inhibits T cell activation. J Immunol.

[B39] Valverde P, Kawai T, Taubman MA (2004). Selective blockade of voltage-gated potassium channels reduces inflammatory bone resorption in experimental periodontal disease. J Bone Miner Res.

[B40] Beeton C, Wulff H, Barbaria J, Clot-Faybesse O, Pennington M, Bernard D, Cahalan MD, Chandy KG, Béraud E (2001). Selective blockade of T lymphocyte K(+) channels ameliorates experimental autoimmune encephalomyelitis, a model for multiple sclerosis. Proc Natl Acad Sci USA.

[B41] Keramidas A, Kuhlmann L, Moorhouse AJ, Barry PH (1999). Measurement of the limiting equivalent conductivities and mobilities of the most prevalent ionic species of EGTA (EGTA2- and EGTA3-) for use in electrophysiological experiments. J Neurosci Methods.

